# Exhaled metabolic markers and relevant dysregulated pathways of lung cancer: a pilot study

**DOI:** 10.1080/07853890.2022.2048064

**Published:** 2022-03-09

**Authors:** Yingchang Zou, Yanjie Hu, Zaile Jiang, Ying Chen, Yuan Zhou, Zhiyou Wang, Yu Wang, Guobao Jiang, Zhiguang Tan, Fangrong Hu

**Affiliations:** a School of Electronic Information and Electrical Engineering, Changsha University, Changsha, China; b Hunan Engineering Technology Research Center of Optoelectronic Health Detection, Changsha, China; c Department of Medicine, Zhejiang Sir Run Run Shaw Hospital, Zhejiang University, Hangzhou, China; d Tianhe Culture Chain Technologies Co Ltd, Changsha, China; e Zhijiang Lab, Research Center for Healthcare Data Science, Hangzhou, China

**Keywords:** Exhaled metabolic markers, volatile organic compounds, pathway enrichment, lung cancer, metabolomics

## Abstract

**Introduction:**

The clinical application of lung cancer detection based on breath test is still challenging due to lack of predictive molecular markers in exhaled breath. This study explored potential lung cancer biomarkers and their related pathways using a typical process for metabolomics investigation.

**Material and methods:**

Breath samples from 60 lung cancer patients and 176 healthy people were analyzed by GC-MS. The original data were GC-MS peak intensity removing background signal. Differential metabolites were selected after univariate statistical analysis and multivariate statistical analysis based on OPLS-DA and Spearman rank correlation analysis. A multivariate PLS-DA model was established based on differential metabolites for pattern recognition. Subsequently, pathway enrichment analysis was performed on differential metabolites.

**Results:**

The discriminant capability was assessed by ROC curve of whom the average AUC and average accuracy in 100-fold cross validations were 0.871 and 0.787, respectively. Eight potential biomarkers were involved in a total of 18 metabolic pathways. Among them, 11 metabolic pathways have *p*-value smaller than .1.

**Discussion:**

Some pathways among them are related to risk factors or therapies of lung cancer. However, more of them are dysregulated pathways of lung cancer reported in studies based on genome or transcriptome data.

**Conclusion:**

We believe that it opens the possibility of using metabolomics methods to analyze data of exhaled breath and promotes involvement of knowledge dataset to cover more volatile metabolites.

**Clinical significance:**

Although a series of related research reported diagnostic models with highly sensitive and specific prediction, the clinical application of lung cancer detection based on breath test is still challenging due to disease heterogeneity and lack of predictive molecular markers in exhaled breath. This study may promote the clinical application of this technique which is suitable for large-scale screening thanks to its low-cost and non-invasiveness. As a result, the mortality of lung cancer may be decreased in future.

**Key messages**
1. In the present study, 11 pathways involving 8 potential biomarkers were discovered to be dysregulated pathways of lung cancer.
2. We found that it is possible to apply metabolomics methods in analysis of data from breath test, which is meaningful to discover convinced volatile markers with definite pathological and histological significance.

## 1. Introduction

Lung cancer is one of the malignant pulmonary diseases that poses a great threat to the health and life of the population. It contributes the highest morbidity rate (11.6% of the total cases) and mortality rate (18.4% of the total cancer death) among all cancers [1]. Owing to lack of typical symptoms, most lung cancer patients are diagnosed at a terminal stage and miss the best treatment period [2]. Screening by the markers that are biologically related to tumour progression can probably provide even more mortality reduction by finding suspected patients as early as possible.

Exhaled breath test is a promising technique for large-scale screening of high-risk population of lung cancer for its convenience, low-cost and non-invasiveness. Therefore, detection of lung cancer through breath test has been thrown into a sharp focus. In 2019, Rudnicka et al. analyzed breath samples from 108 patients with lung cancer and 121 healthy volunteers with chromatography-mass spectrometry (GC-MS) [3]. Cross-validation of the obtained model has shown the sensitivity of 80% and specificity of 91.23%. In addition, Huang and Li used selected ion flow tube-mass spectrometry (SIFT-MS) technique to quantitatively analyze 116 volatile organic compounds (VOCs) in breath samples from 148 patients with histologically confirmed lung cancers and 168 healthy volunteers. A diagnostic model based on eXtreme Gradient Boosting (XGBoost) method was built, showing accuracy of 92% [4]. Although a series of research reported diagnostic models with highly sensitive and specific prediction, far exceeding the performance of currently available low-dose computed tomography (LDCT) detection, the clinical application of this technique is still challenging due to disease heterogeneity and lack of predictive molecular markers in exhaled breath.

Recently, Chen et al. used GC-MS data from 160 patients with lung cancer, 70 patients with benign pulmonary disease and 122 healthy subjects to exploring exhaled markers of lung cancer. As a result, they found that 20 VOCs discriminated lung cancer from healthy subjects. Additionally, their reported 19 and 20 VOCs related to histological type and lung cancer stages, respectively [5]. Actually, many efforts have been made selecting a series of breath markers associated with lung cancer [6,7] since 1985 [8]. However, none of them have been used in clinical because the underlying mechanisms about how those VOCs produced are still unclear.

Metabolomics works as a multidiscipline crossed diagnostic tool for exploring differences and dynamic changes in endogenous micromolecular metabolites, combining analytical chemistry and bioinformatics to systematically detect and analyze changes in metabolites in the body. It has been widely used in diverse metabolic samples including urine [9,10], serum/plasma [11,12] and tissue [13,14]. Based on those high-throughput data and online metabolic database, e.g. the human metabolome database (HMDB) and kyoto encyclopaedia of genes and genomes (KEGG), pathway enrichment analysis can be done, which may explain the relationship between metabolic data and pathophysiological state. As a mature technique, metabolomics provides a scientific and systematic data mining process for differential metabolites analysis, ensuring validity, interpretability, and reproducibility of their results. However, few studies analyzed breath data with metabolic database, even though there are various software and tools designed for guiding and performing metabolomics data analysis.

Here, we are going to identify molecular markers of metabolic dysregulation in lung cancer using the GC-MS data obtained from a recent study [15]. In the mentioned study, we reported a method to differentiate subjects with lung cancer from healthy controls, by means of exhaled breath test with the GC-MS. Then, breath profiles instead of volatile markers were analyzed with machine-learning algorithm and an accuracy of 85% was shown in six-fold cross validations. Although that study does relatively well in diagnosis, there is still necessity to illustrate the combination of metabolic markers and their relative pathways before application in clinic. Pathway enrichment analysis is a knowledge-based approach, depending largely on databases available for bioinformatic analysis such as KEGG and HMDB. Accordingly, our statistical analysis focussed on those candidate VOCs which could be annotated to online databases. This study was performed not only to find potential markers and relevant pathways for detection of lung cancer but also to explore the possibility of combination of metabolomics methods and breath data.

## 2. Method

### 2.1. Data acquisition

Data were obtained from a previous case–control study where 236 subjects were asked to participate [15]. All cases were confirmed with an incident of lung cancer histologically or pathologically, while controls were confirmed with a negative result of LDCT scan. The detailed inclusion criteria and exclusion criteria of subjects were listed in Supplementary materials (S1).

Sample collection and analysis were performed as previously published [15]. Briefly, to collect breath samples, subjects were asked to breathe tidally into a self-developed collection device with which VOCs in 1000 mL exhaled breath were captured and concentrated into a Tenax TA stainless steel tube (PerkinElmer, Waltham, MA). Then, each sampling tube was shipped to laboratory for chemical analysis which was performed on GC-MS (QP2010 Plus, Shimadzu, Tokyo, Japan) coupled with a thermal desorption (TD) instrument (TurboMatrix 300 TD, PerkinElmer, Waltham, MA). Subsequently, spectrum analysis including peak identification and background removal was done. Details of collection, detection and data pre-treatment are illustrated in Supplementary materials (S2). Metabolites which can be annotated to HMDB were then used for following analysis.

### 2.2. Statistical analysis

Statistical analysis was applied to VOCs present in more than 70% of samples in at least one group, with a quality control relative standard deviation smaller than 25%. A metabolite is described as “putative” following an accurate mass match to the HMDB database [16].

Univariate statistical analysis was performed on filtered data using the Mann–Whitney test. VOCs with fold change larger than 2 and FDR *p* < .1 were selected as candidate differential metabolic biomarkers. Due to the confounding effects of smoking and gender, stratification by smoking status and gender were applied in univariate statistical analysis. In five times of group-wise Mann–Whitney tests, VOCs selected as differential metabolic biomarkers at least once were employed in following analysis.

Before multivariate analysis, data of all significantly altered metabolites were cube root-transformed, mean-centred and divided by the standard deviation of each variable. Orthogonal projections to latent structures discriminant analysis (OPLS-DA) and spearman rank correlation analysis were performed on scaled data.

Those confirmed metabolites were imported into MBRole 2.0 for pathway enrichment analysis.

Statistical analysis and data visualization are implemented by Metaboanalyst (www.metaboanalyst.ca/) and GraphPad-Prism 7.0 software (GraphPad Software, La Jolla, CA), except for Upset plot (https://www.omicstudio.cn/tool) and enrichment analysis (http://csbg.cnb.csic.es/mbrole2/). The sample size per group was confirmed to be effective through power analysis on Metaboanalyst before any other statistical analysis (S3).

## 3. Results

### 3.1. Subjects

Two hundred thirty-six participants including 60 lung cancer patients and 176 healthy subjects were recruited in the study (Table 1). There were no significant differences of smoking history and age between two groups. Unfortunately, the moderate imbalance of genders existed when the significance level was set as 0.05. Considering univariate statistical analysis were conducted for males and females, respectively, the differences with *p*-value of .024 were acceptable.

**Table 1.** Clinical characteristics between case and control groups.

		Lung cancer		Health		*p*-value	
		(*N* = 60)		(*N* = 176)			
Gender							
Male		37		135		.024^#^	
Female		23		41			
Smoking history							
Smoker		32		112		.096^#^	
Non-smoker		28		64			
Age (mean, SD)		62.4, 10.514		64.53, 13.252		.158*	
Adenocarcinoma/ squamous cell carcinoma		32, 20					
Stage I/II/III/IV		14, 6, 19, 21					
Comorbidities							
Type 2 diabetes mellitus	4		8		.507		
Systemic arterial hypertension	6		11		.386		

^#^Chi-square tests.

*Independent-samples Mann–Whitney *U* tests.

### 3.2. Univariate statistical analysis

**Figure 1.**
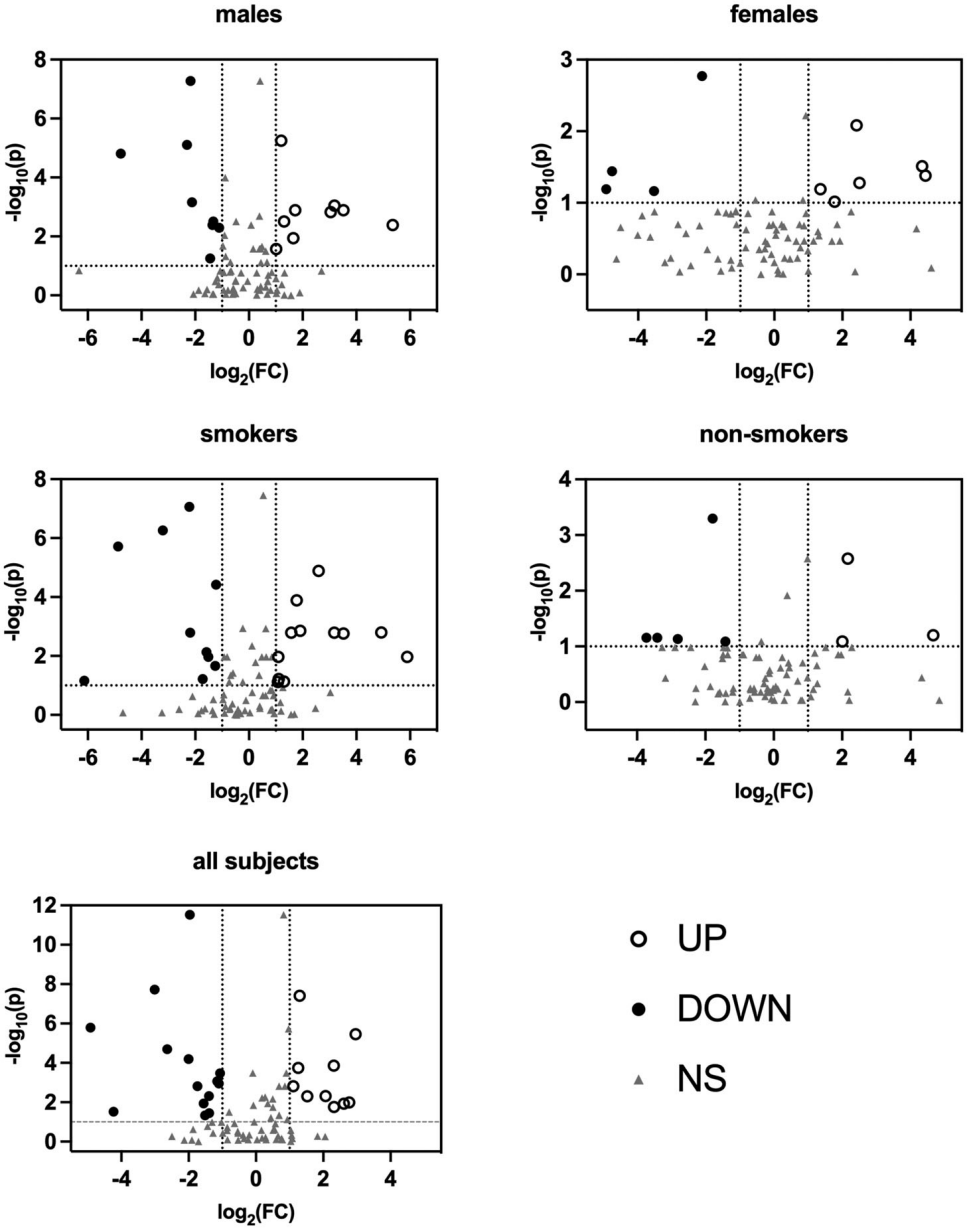
Volcano plots of Mann–Whitney test data. Black circles (UP) represent metabolites upregulated in patients with lung cancer; black points (DOWN) represent metabolites downregulated in patients with lung cancer; gray triangles (NS) represent metabolites has no statistical significance in Mann-Whitney test.

A total of 308 VOCs were obtained after data pre-treatment, of whom 81 had confirmed HMDB annotations, namely the putative metabolites. Volcano plots displaying log-fold-change of signal against *p*-value from non-parametric test were employed to show the results of univariate statistical analysis [17] (Figure 1). According to the selection criteria ((fold change) > 2, (FDR p) < .1), several differential metabolites were selected in each group, respectively. The fold change data and the corresponding FDR *p*-values of group-wise differential metabolites were listed in Supplementary materials (S4).

**Figure 2.**
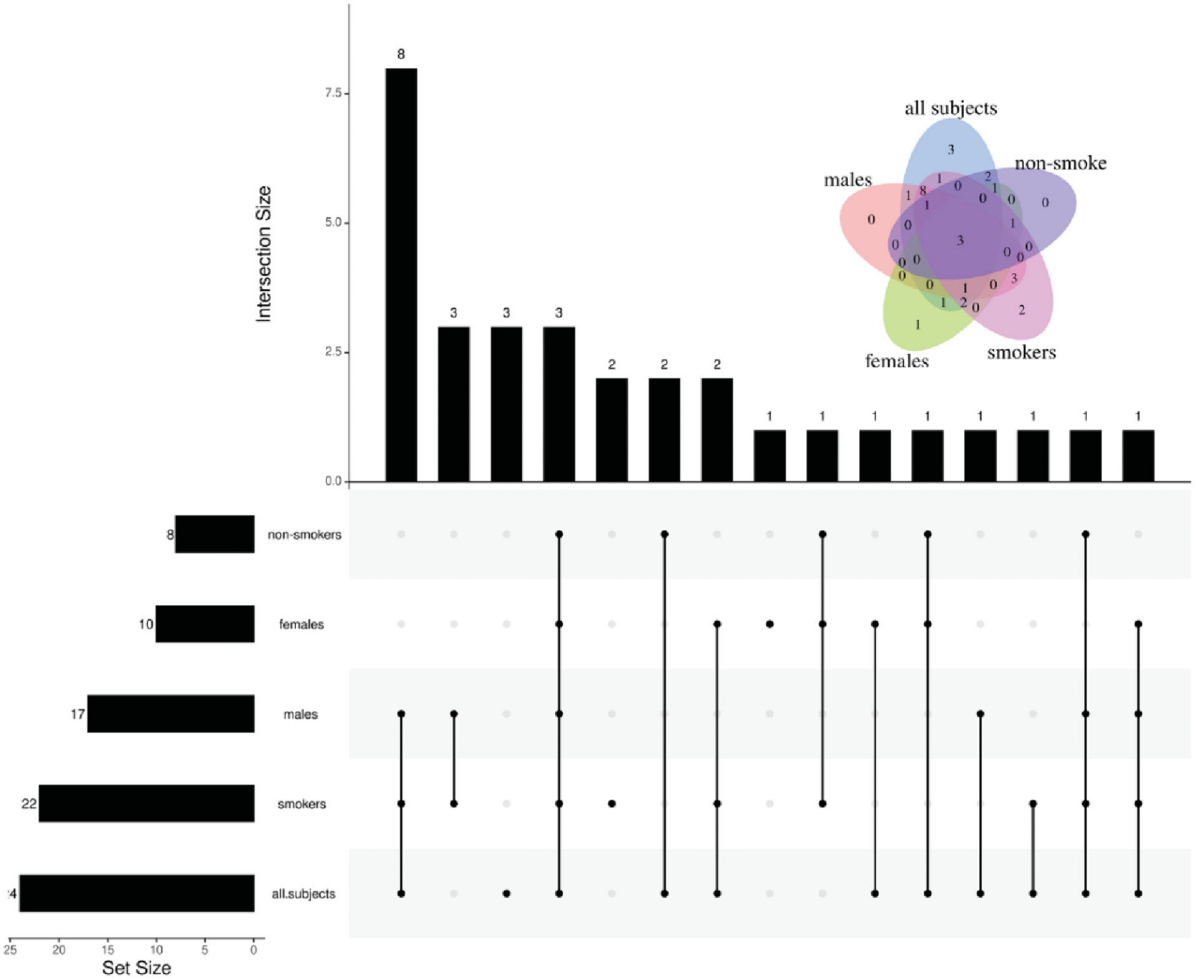
Upset plot of the candidate differential metabolites.

In addition to comparison stratified by genders and smoking status, the univariate statistical analysis was also performed on all subjects. Twenty-four VOCs were found to be differential metabolites (Figure 1 and Figure S4), of whom 10 upregulated in patients, while others downregulated. As shown in Figure 2, there were 8, 10, 7, 22 and 24 kinds of metabolites selected in males, females, smokers, non-smokers and all subjects, respectively. A total of 31 VOCs presenting at least once among group-wise differential metabolites were determined as candidate differential metabolites (Table 2).

**Table 2.** Candidate differential metabolites.

HMDB	Match	PubChem	CAS	KEGG
HMDB0031447	n-Heptane	8900	142-82-5	NA
HMDB0033182	2,5-Dimethylfuran	12266	625-86-5	NA
HMDB0034237	Propyl acetate	7997	109-60-4	NA
HMDB0031653	3-(Methylthio)-1-propene	66282	10152-76-8	NA
HMDB0005879	Dimethyldisulfide	12232	624-92-0	C08371
HMDB0031583	3-Methylheptane	11519	589-81-1	NA
HMDB0005994	Hexanal	6184	66-25-1	C02373
HMDB0031325	n-Butylacetate	31272	123-86-4	C12304
HMDB0059851	o-Xylene	7237	95-47-6	C07212
HMDB0000042	Acetic acid	176	64-19-7	C00033
HMDB0029595	n-Nonane	8141	111-84-2	C02445
HMDB0034029	Isopropylbenzene	7406	98-82-8	C14396
HMDB0059839	Camphene	6616	79-92-5	C06076
HMDB0059848	3-Ethyltoluene	12100	620-14-4	C14522
HMDB0001140	n-Octanal	454	124-13-0	C01545
HMDB0035619	3-Carene	26049	13466-78-9	NA
HMDB0005805	p-Cymene	7463	99-87-6	C06575
HMDB0032473	Limonene	22311	138-86-3	C06078
HMDB0031445	n-Undecane	14257	1120-21-4	NA
HMDB0059835	n-Nonanal	31289	124-19-6	NA
HMDB0003352	l-Menthol	16666	2216-51-5	C00400
HMDB0002019	Phytol	5280435	150-86-7	C01389
HMDB0032860	1-Methylnaphthalene	7002	90-12-0	C14082
HMDB0006007	3-Methylbutanol	31260	123-51-3	C07328
HMDB0032449	1-Octene	8125	111-66-0	NA
HMDB0031231	2-Ethylhexanol	7720	104-76-7	C02498
HMDB0004472	Eucalyptol	2758	470-82-6	C09844
HMDB0037050	o-Cymene	10703	527-84-4	NA
HMDB0031418	3,3-Dimethylhexane	11233	563-16-6	NA
HMDB0001183	n-Octanol	957	111-87-5	C00756
HMDB0031327	2-Butoxyethanol	8133	111-76-2	C19355

### 3.3. Multivariate statistical analysis

**Figure 3.**
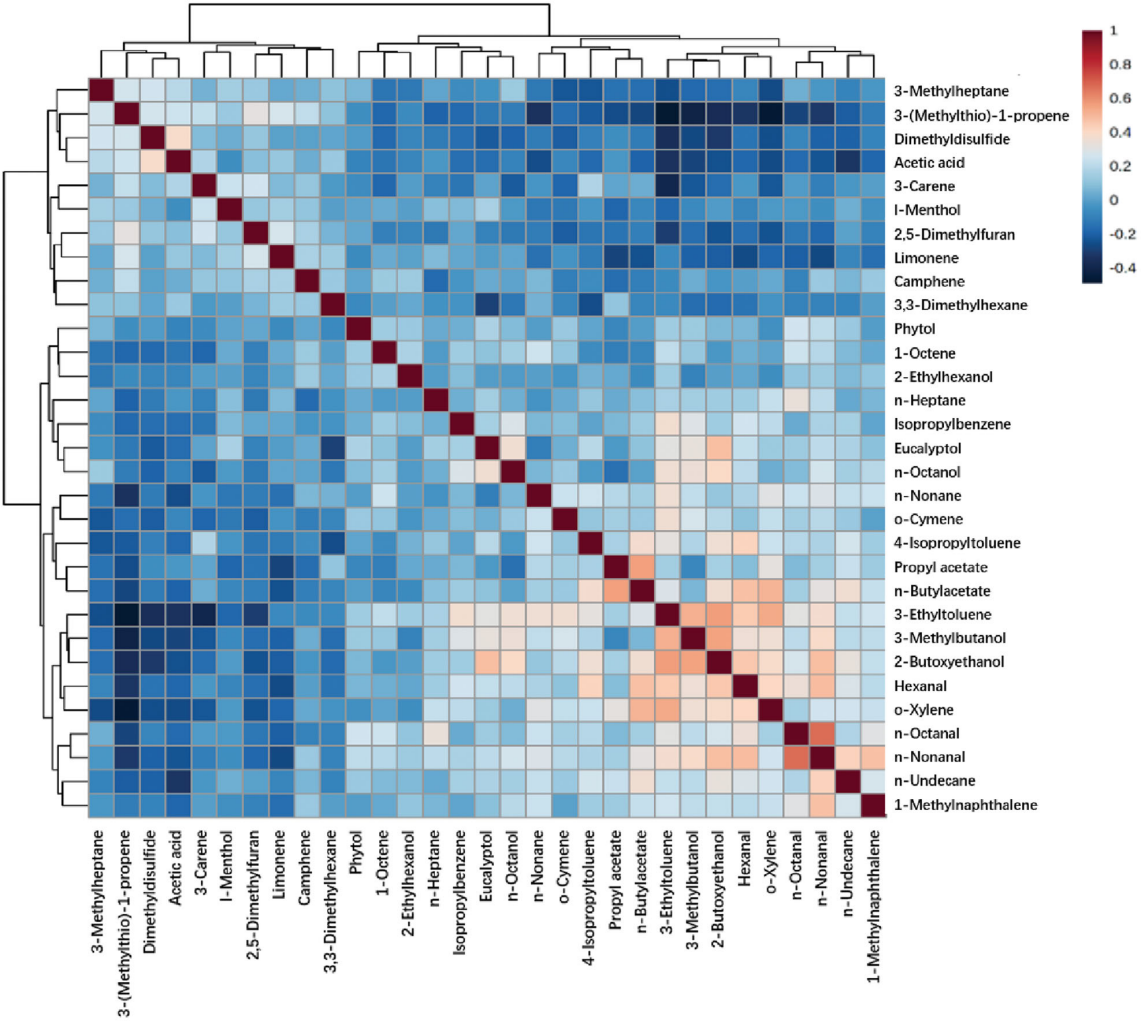
Spearman rank correlation analysis among 31 selected metabolites.

Correlation analyses were performed for all candidate differential metabolites with each other using Spearman’s correlation (Figure 3 and Figures S5 and S6). N-Nonanal and n-Octanal has the strongest correlation among all metabolites (*r* = 0.67426, *p* < .001). Besides them, there were seven pairs of metabolites having correlation coefficients larger than 0.5 (S7). The rests of correlation coefficients were smaller than 0.5. It could be concluded that there is no strong correlation among all VOCs based on reference [18]. Thus, it is not necessary to remove any one from these 31 VOCs.

**Figure 4.**
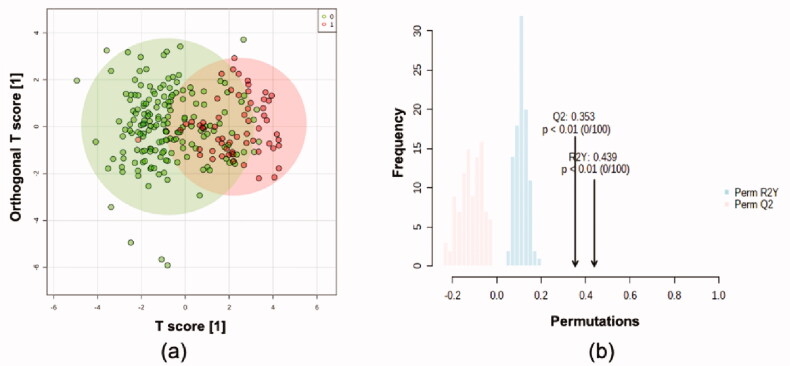
Orthogonal projections to latent structures discriminant analysis based on 236*31 dataset. (a) Score plot. Green points representing controls while red points representing cases. (b) Permutation tests.

Multivariate statistical modelling was performed using OPLS-DA on the 31 confirmed metabolites. This model showed moderately significant group separation (*Q*^2^=0.331, *R*^2^*Y* = 0.357, Figure 4(a)). Permutation tests confirmed the robustness of the model (100 permutations, *Q*^2^=0.353, *R*^2^Y = 0.439, Figure 4(b)). PLS-DA was also performed, and the score plots are shown in Supplementary materials (S8).

**Figure 5.**
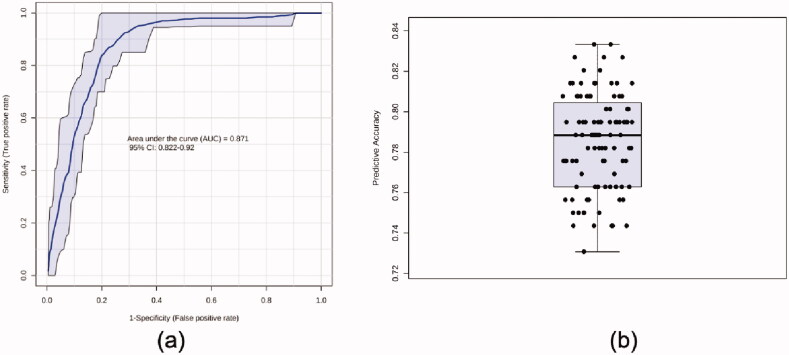
ROC analysis based on PLS-DA. (a) ROC curve and (b) predictive accuracy in 100 cross validations.

As subjects from case and control groups cannot be separated completely in OPLS-DA, ROC analysis was performed on output values of PLS-DA (Figure 5). Areas under the curve (AUCs) ranged from 0.822 to 0.92 in 100 cross validations, and the average predictive accuracy was 0.787.

**Figure 6.**
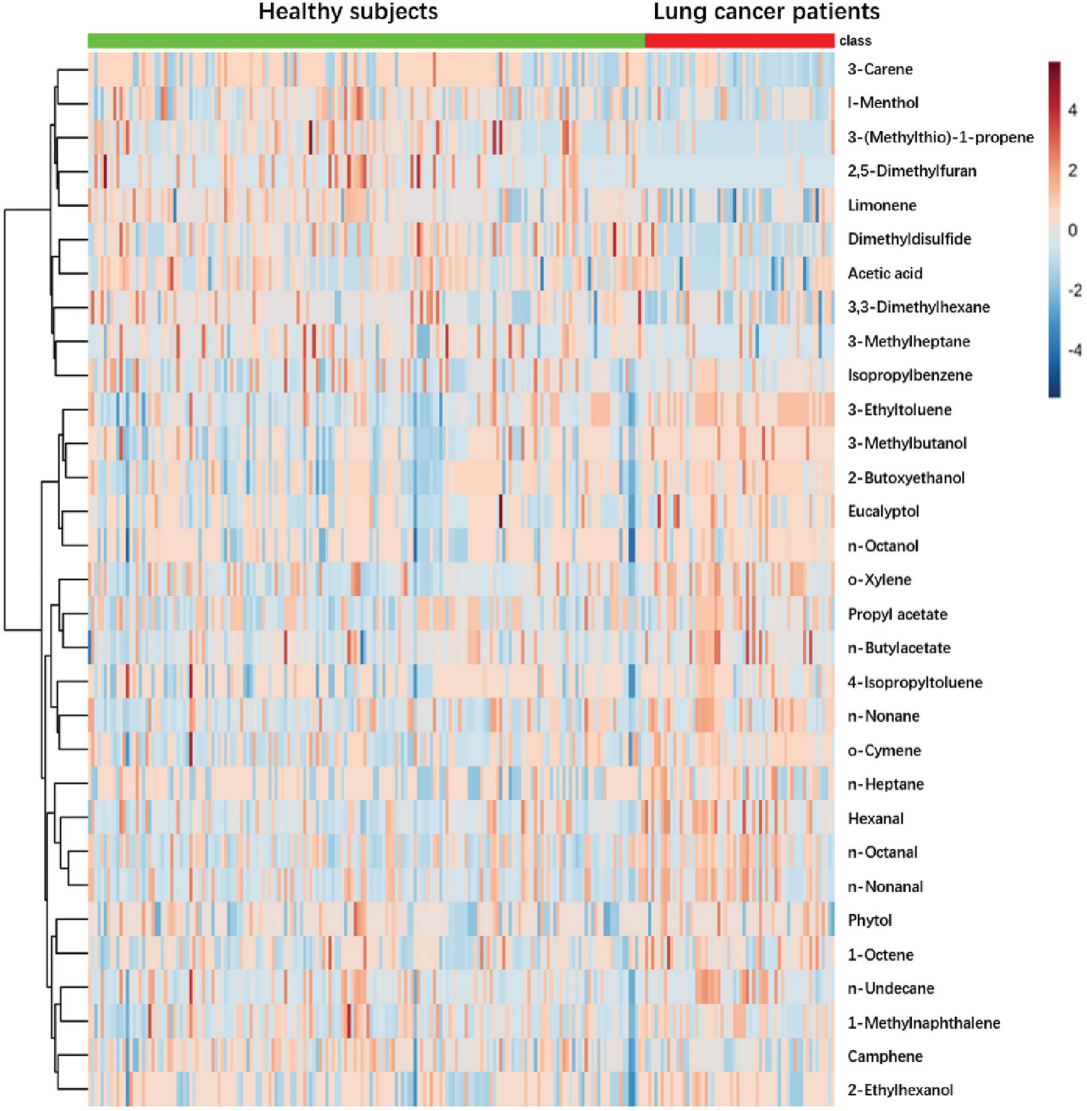
A heatmap of differential metabolite profiles of 236 samples.

Scaled peak intensity of 31 differential metabolites from 60 cases and 176 controls were displayed in a heatmap (Figure 6), showing that each group had its specific metabolic profiles. In detail, for metabolites lying in upper rows, patients have relatively lower levels than those of controls. For metabolites lying in lower rows, the situation was opposite.

### 3.4. Pathway enrichment

**Figure 7.**
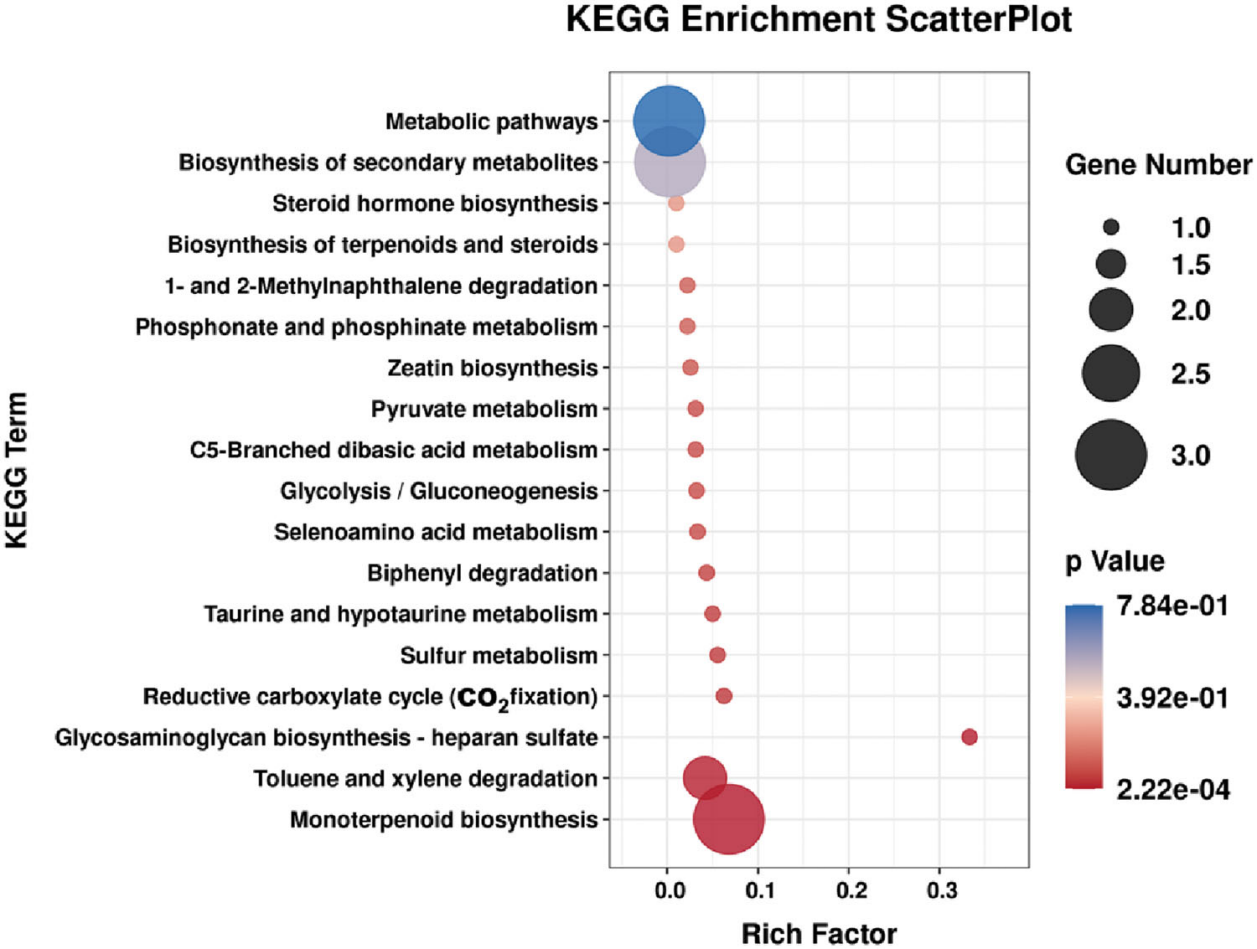
Bubble plot of the metabolic pathway.

To further explore the relationship between the above 31 differential metabolites and the pathogenesis of lung cancer, these small molecular metabolites were introduced into MBRole 2.0 (http://csbg.cnb.csic.es/mbrole2/) to obtain the key metabolic pathways involved. As shown in Figure 7 and Table 3, 8 potential biomarkers (Table 4) were involved in a total of 18 metabolic pathways. Among them, 11 metabolic pathways have *p*-value smaller than .1, indicating that they have significant contribution to the lung cancer metabolic pathway, namely monterpenoid biosynthesis, toluene and xylene degradation, glycosaminoglycan biosynthesis-heparan sulphate, reductive carboxylate cycle (CO_2_ fixation), biphenyl degradation, glycolysis/gluconeogenesis, C5-branched dibasic acid metabolism, pyruvate metabolism, selenoamino acid metabolism, taurine and hypotaurine metabolism and sulphur metabolism. Wherein, glycosaminoglycan biosynthesis - heparan sulphate has the greatest rich factor of 0.3333.

**Table 3.** Pathway enrichment analysis based on differential metabolites.

KEGG ID annotation	Annotation	Rich factor	*p*-value	Matching IDs
map00902	Monoterpenoid biosynthesis	0.0682	.00022228	C00400
				C06076
				C09844
map00622	Toluene and xylene degradation	0.0417	.00750019	C00033
				C07212
map00534	Glycosaminoglycan biosynthesis-heparan sulphate	0.3333	.00818327	C00033
map00720	Reductive carboxylate cycle (CO_2_ fixation)	0.0625	.04290716	C00033
map00621	Biphenyl degradation	0.0435	.06111813	C06575
map00010	Glycolysis / Gluconeogenesis	0.0323	.08152337	C00033
map00660	C5-Branched dibasic acid metabolism	0.0313	.08404388	C00033
map00620	Pyruvate metabolism	0.0313	.08404388	C00033
map00450	Seleno-amino acid metabolism	0.0333	.07899619	C00033
map00430	Taurine and hypotaurine metabolism	0.0500	.05335453	C00033
map00920	Sulphur metabolism	0.0556	.04814461	C00033
map00440	Phosphonate and phosphinate metabolism	0.0222	.11621235	C00033
map00908	Zeatin biosynthesis	0.0256	.1015025	C00033
map00624	1- and 2-Methylnaphthalene degradation	0.0222	.11621235	C14082
map00140	Steroid hormone biosynthesis	0.0101	.23859889	C02373
map01062	Biosynthesis of terpenoids and steroids	0.0102	.23648811	C06076
map01110	Biosynthesis of secondary metabolites	0.0029	.55196064	C00033
				C00400
				C06076
map01100	Metabolic pathways	0.0021	.78425283	C00033
				C06076
				C07212

**Table 4.** Potential biomarkers of lung cancer in exhaled breath.

Name	HMDB	PubChem	KEGG	CAS
Hexanal	HMDB0005994	6184	C02373	66-25-1
o-Xylene	HMDB0059851	7237	C07212	95-47-6
Acetic acid	HMDB0000042	176	C00033	64-19-7
Camphene	HMDB0059839	6616	C06076	79-92-5
p-Cymene	HMDB0005805	7463	C06575	99-87-6
Menthol	HMDB0003352	16666	C00400	2216-51-5
1-Methylnaphthalene	HMDB0032860	7002	C14082	90-12-0
Eucalyptol	HMDB0004472	2758	C09844	470-82-6

## 4. Discussion

Although 308 kinds of VOCs were detected, the number of putative metabolites used to analysis is only 81. Many volatile metabolites were not able to be annotated in HMDB or KEGG database likely account for this remarkable difference between the number of detected VOCs and the number of putative metabolites. As a knowledgebase of human metabolome, HMDB involves a series recording of metabolites derived from human serum, urine, saliva and so on. Although HMDB has been continuously improving in the past decades, the exhaled breath as one of metabolic products of human body has not been included. It reflects metabolomic studies on breath test are still in its infancy. Although VOCs in breath has been detected since 1971, there are still lots of mystery compounds in exhaled breath, especially those enzymatically and non-enzymatically transformed products derived from well-known endogenous or exogenous compounds. As consequent, HMDB are not providing the necessary metabolite coverage to allow researchers to identify these VOCs in breath.

Totally, 31 kinds of differential metabolites were selected from 81 putative metabolites, through the Mann–Whitney test, Spearman rank correlation analysis and OPLS-DA. In cross-validation, the average accuracy of the multivariate model based on these 31 VOCs is 78.7%, while that of the previously reported model selecting VOCs by machine learning algorithm was 85% [15]. In our opinion, these differences were derived from that some VOCs which may be valuable for pattern recognition were removed unexpectedly during the process of HMDB annotations. Obviously, those removal were related to the lack of available data and knowledge on breath research in HMDB database. However, the main intention of this study is to explore several volatile biomarkers and related pathways instead of overemphasizing the pursuit of accuracy. As we mentioned before, diagnostic models separated from biomedical meanings are always not robust enough. So, it is significantly meaningful to obtain several kinds of confident markers, even though that is not the entire set of lung cancer markers in breath.

Eleven pathways involving eight potential biomarkers were discovered in enrichment analysis. Among them, monoterpenoid biosynthesis pathway has the lowest *p*-value, indicating the statistical significance. Menthol, camphene and eucalyptol were annotated in this pathway. First, monoterpenoid chemicals were sometimes used in treatment [19–21]. For instance, camphene was reported to be a main component of essential oils of lemongrass which induces apoptosis and cell cycle arrest in A549 lung cancer cells [22]. In addition, eucalyptol shows several pharmacologic activities that may be used in treatment of some pulmonary disease including rhinosinusitis, bronchitis, asthma and chronic obstructive pulmonary disorder (COPD) [23]. Likewise, camphene could be beneficial in battling respiratory illnesses, and could act as a cough suppressant and anti-congestive tool [24]. Indeed, patients who received treatment after the diagnosis of LC or had a history of airway inflammatory in the past 3 months were excluded. However, inclusion or exclusion of subjects were based on medical record in our hospital and questionnaire survey. Questionnaire survey, the criteria of every patient are obviously subjective. Generally speaking, lung cancer always comes with pulmonary symptoms. Therefore, a large number patient may dose themselves with some cough suppressant or cold drug and did not state in their questionnaire. However, it could have been avoided to a certain extent by more careful and scientific design of questionnaire. In future research, we would give more related examples and more strict definition of each symptom and treatment. Secondly, some cigarettes may contain menthol, and menthol cigarettes has been confirmed to increase lung cancer risk [25,26]. So, it is not sure whether the regulation of monoterpenoid biosynthesis pathway is related to lung cancer or other factors including therapy and smoking. Although their relation to lung cancer is not yet clear, menthol, camphene and eucalyptol were detected when comparing breath VOCs from smokers and non-smokers with and without COPD [27]. This literature also suggested even though some VOCs relate not only to disease but also to smoking status, detections of their concentrations still make sense for disease diagnosis.

As regard to toluene and xylene degradation, o-xylene and acetic acid were found in this pathway. Although we did not find any literature reporting the relations of this pathway and lung cancer incidence, toluene and xylene are confirmed to be risk factor of lung cancer [28,29]. Additionally, they have been detected in exhaled breath [30], especially they were reported as lung cancer markers in exhaled breath in a series of papers [31–34]. But many aromatic VOCs were also reported as results of cigarettes exposure [35], which made it doubtful whether they can be available lung cancer markers. In our view, even cigarette smoke may be the source of these molecules, their levels still make sense. Because the key point is the difference of degradation capacity rather than absolute concentrations of those molecules. To test our hypothesis, we applied univariate statistical analysis in people with different genders and smoking status. Group-wise differential metabolites were listed in Supplementary materials (S4) which showed that o-xylene was selected as differential metabolite both in smokers and non-smokers. However, it has no significant differences between subjects with and without lung cancer, when we took all subjects including smokers and non-smokers into consideration. Similar results were shown in a dual centre study comparing breath VOCs which was mentioned above [27]. In that study, COPD patients were diagnosed from smokers and non-smokers, respectively, to overcoming the confounding effects of smoking. However, the sample size of each subgroup was limited. More longitudinal studies for aromatic VOCs should be conducted in future, especially focussing on toluene and xylene degradation pathway.

Other pathways obtained in our study were reported as lung cancer-related pathway before, including glycosaminoglycan biosynthesis-heparan sulphate, reductive carboxylate cycle (CO_2_ fixation), glycolysis/gluconeogenesis, C5-branched dibasic acid metabolism, pyruvate metabolism, selenoamino acid metabolism, taurine and hypotaurine metabolism and sulphur metabolism. Although metabolism analysis such as pathway enrichment were rarely used in studies on lung cancer biomarkers in breath, similar studies have been conducted based on miRNA or DNA data. That provides lots of meaningful information for our work. Higher impact values indicated that these metabolic pathways are more relevant to the pathogenesis of lung cancer. Among all pathways, we enriched, glycosaminoglycan biosynthesis-heparan sulphate with rich factor of o.333 (*p* = .008) may be the most possible dysregulated pathway related to lung cancer. Yang et al. reported it as one of the biologic pathways enriched by differentially expressed smoking and lung cancer specific miRNA [36]. Similarly, reductive carboxylate cycle (CO_2_ fixation) was reported as lung cancer related pathway in 2016 [37]. Wang et al. investigated specific genotypes of different subtypes or stages of lung cancer through gene expression variations of chromosome 2 genes. IDH1 were selected as differential gene and enriched to reductive carboxylate cycle (CO_2_ fixation) pathway which is upregulated in lung cancer. Huang et al. performed a meta-analysis of 4 lung cancer microarray datasets encompassing 353 patients to reveal differentially expressed genes between normal lung tissues and lung cancer of different stages [38]. Overall, 1838 genes were found to be dysregulated. glycolysis/gluconeogenesis were showed to be one of significantly regulated pathway in lung cancer. As regard to C5-branched dibasic acid metabolism, a genome-scale metabolic models for exploring changes in metabolism under normal and cancer conditions have concluded this pathway is relevant to lung cancer and prostate cancer [39]. Additionally, its dysregulation is also related to cystic fibrosis which is one of the risk factor of lung cancer [40]. As with pathways above, pyruvate metabolism [41,42], seleno-amino acid metabolism [43] taurine and hypotaurine metabolism [44] also be confirmed to be closely related to lung cancer through other kinds of omics data.

Although we believe that abnormalities of VOCs in breath of lung cancer patients are closely related to dysregulation of these pathways, results are not convinced enough. More details about relationship between lung cancer and volatile metabolites, like genetic-level information, are necessary. Other kinds of omic data have also been involved in HMDB, which made it more compatible with the increasing number of multi-omic or systems biology studies [16]. Recently, efforts have been made on searching lung cancer markers in exhaled breath condensate (EBC) where genes [45] and proteins [46] could be detected. With different sampling technique, multi-omics data including VOCs, genes and proteins could be acquired in exhaled breath, simultaneously. Therefore, studies combining volatile breath and EBC may be a promising way to do some data mining on exhaled lung cancer markers and their related pathways.

There still some limitations in this study. As far as we known, subtypes and stages of lung cancer have influences on metabolic disorders. However, the sample size in our study is not enough for comparisons among subgroups. Other limitation is that too many VOCs were not involved in HMDB database, owing to few studies on pathway related to volatile metabolites. Therefore, metabolomics cannot work in exploring breath marker as well as it should be. Further, lack of standards of sampling techniques and analytical techniques may lead to different results between various studies. For instance, expiratory flow rate, breath hold and inclusion of anatomical dead space were reported to be significant influence factors of breath test [47]. Addressing these weaknesses requires more researchers making efforts to expand the metabolic dataset, and standardize the sampling and analytical techniques.

## 5. Conclusion

A key challenge for efforts to apply breath diagnosis of lung cancer in clinical is the lack of clear explanation about relationship between volatile makers and lung cancer. Although our study failed to provide a list of all markers in breath, we still open the possibility of exploring dysregulated pathway which result in variation of VOCs in breath, which may illustrate where these markers derived from. We believe that with the gradually improved bioinformatic database (e.g. HMDB or KEGG) the bottleneck of studies on exhaled markers of lung cancer may be removed.

## Supplementary Material

Supplemental Material

## Data Availability

The data based on the results of the current study were obtained, are accessible from the corresponding authors upon reasonable request.

## Supplementary materials

S1: Inclusion criteria and exclusion criteria, S2: Details of data acquisition, S3: Power analysis plot, S4: Group-wise differential metabolites, S5: Correlation coefficients of correlation analysis, S6: *p*-values of correlation analysis, S7: Correlations of metabolites larger than 0.5, S8: PLS-DA score plots.

## References

[1] Bray F, Ferlay J, Soerjomataram I, et al. Global cancer statistics 2018: GLOBOCAN estimates of incidence and mortality worldwide for 36 cancers in 185 countries. CA Cancer J Clin. 2018;68(6):394–424. 30207593 10.3322/caac.21492

[2] Siegel RL, Miller KD, Jemal A. Cancer statistics, 2016. CA Cancer J Clin. 2016;66(1):7–30. 26742998 10.3322/caac.21332

[3] Rudnicka J, Kowalkowski T, Buszewski B. Searching for selected VOCs in human breath samples as potential markers of lung cancer. Lung Cancer. 2019;135:123–129. 31446984 10.1016/j.lungcan.2019.02.012

[4] Tsou P-H, Lin Z-L, Pan Y-C, et al. Exploring volatile organic compounds in breath for high-accuracy prediction of lung cancer. Cancers. 2021;13(6):1431. 33801001 10.3390/cancers13061431 PMC8003836

[5] Chen X, Muhammad KG, Madeeha C, et al. Calculated indices of volatile organic compounds (VOCs) in exhalation for lung cancer screening and early detection. Lung Cancer. 2021;154:197–205. 33653598 10.1016/j.lungcan.2021.02.006

[6] Ratiu IA, Ligor T, Bocos-Bintintan V, et al. Volatile organic compounds in exhaled breath as fingerprints of lung cancer, asthma and COPD. JCM. 2020;10(1):32. 10.3390/jcm10010032 PMC7796324 33374433

[7] Janssens E, van Meerbeeck JP, Lamote KJCRiOH. Volatile organic compounds in human matrices as lung cancer biomarkers: a systematic review. Crit Rev Oncol/Hematol. 2020;153:103037. 10.1016/j.critrevonc.2020.103037 32771940

[8] Gordon SM, Szidon JP, Krotoszynski BK, et al. Volatile organic compounds in exhaled air from patients with lung cancer. Clin Chem. 1985;31(8):1278–1282. 4017231

[9] Amin AM, Mostafa H, Arif NH, et al. Metabolomics profiling and pathway analysis of human plasma and urine reveal further insights into the multifactorial nature of coronary artery disease. Clin Chim Acta. 2019;493:112–122. 30826371 10.1016/j.cca.2019.02.030

[10] Liu X, Zhang M, Liu X, et al. Urine metabolomics for renal cell carcinoma (RCC) prediction: tryptophan metabolism as an important pathway in RCC. Front Oncol. 2019;9:663. 31380290 10.3389/fonc.2019.00663 PMC6653643

[11] Suarez-Diez M, Adam J, Adamski J, et al. Plasma and serum metabolite association networks: comparability within and between studies using NMR and MS profiling. J Proteome Res. 2017;16(7):2547–2559. 28517934 10.1021/acs.jproteome.7b00106 PMC5645760

[12] Jasbi P, Wang D, Cheng SL, et al. Breast cancer detection using targeted plasma metabolomics. J Chromatogr B Analyt Technol Biomed Life Sci. 2019;1105:26–37. 10.1016/j.jchromb.2018.11.029 30562627

[13] Vander Heiden M, Lunt SY, Dayton TL, et al. Metabolic pathway alterations that support cell proliferation. In: Cold Spring Harbor symposia on quantitative biology. Cold Spring Harbor: Cold Spring Harbor Laboratory Press; 2011. 10.1101/sqb.2012.76.010900 22262476

[14] Gonzalez-Riano C, Garcia A, Barbas C. Metabolomics studies in brain tissue: a review. J Pharm Biomed Anal. 2016;130:141–168. 27451335 10.1016/j.jpba.2016.07.008

[15] Zou Y, Wang Y, Jiang Z, et al. Breath profile as composite biomarkers for lung cancer diagnosis. Lung Cancer. 2021;154:206–213. 33563485 10.1016/j.lungcan.2021.01.020

[16] Wishart DS, Feunang YD, Marcu A, et al. HMDB 4.0: the human metabolome database for 2018. Nucleic Acids Res. 2018;46(D1):D608–D617. 29140435 10.1093/nar/gkx1089 PMC5753273

[17] Li W. Volcano plots in analyzing differential expressions with mRNA microarrays. J Bioinform Comput Biol. 2012;10(6):1231003. 23075208 10.1142/S0219720012310038

[18] Källén B. Statistics for dummies. In: Drugs during pregnancy. New York, NY: Springer; 2016. p. 77–87.

[19] El Gaafary M, Hafner S, Lang SJ, et al. A novel polyhalogenated monoterpene induces cell cycle arrest and apoptosis in breast cancer cells. Mar Drugs. 2019;17(8):437. 10.3390/md17080437 PMC6723102 31349625

[20] Ponomarev KY, Suslov EV, Zakharenko AL, et al. Aminoadamantanes containing monoterpene-derived fragments as potent tyrosyl-DNA phosphodiesterase 1 inhibitors. Bioorg Chem. 2018;76:392–399. 29248742 10.1016/j.bioorg.2017.12.005

[21] Qiu D, Zhou M, Chen J, et al. Hyperelodiones AC, monoterpenoid polyprenylated acylphoroglucinols from hypericum elodeoides, induce cancer cells apoptosis by targeting RXRα. Phytochemistry. 2020;170:112216. 31841782 10.1016/j.phytochem.2019.112216

[22] Trang DT, Hoang TKV, Nguyen TTM, et al. Essential oils of lemongrass (*Cymbopogon citratus* Stapf) induces apoptosis and cell cycle arrest in A549 lung cancer cells. Biomed Res Int. 2020;2020:5924856. 32420353 10.1155/2020/5924856 PMC7201560

[23] Galan DM, Ezeudu NE, Garcia J, et al. Eucalyptol (1, 8-cineole): an underutilized ally in respiratory disorders? J Essent Oil Res. 2020;32(2):103–110.

[24] Sultana S, Khan A, Safhi MM, et al. Cough suppressant herbal drugs: a review. Int J Pharm Sci Invent. 2016;5(5):15–28.

[25] Brooks DR, Palmer JR, Strom BL, et al. Menthol cigarettes and risk of lung cancer. Am J Epidemiol. 2003;158(7):609–616. 14507595 10.1093/aje/kwg182

[26] Blot WJ, Cohen SS, Aldrich M, et al. Lung cancer risk among smokers of menthol cigarettes. J Natl Cancer Inst. 2011;103(10):810–816. 21436064 10.1093/jnci/djr102 PMC3096798

[27] Gaida A, Holz O, Nell C, et al. A dual center study to compare breath volatile organic compounds from smokers and non-smokers with and without COPD. J Breath Res. 2016;10(2):026006. 27082437 10.1088/1752-7155/10/2/026006

[28] Gérin M, Siemiatycki J, Désy M, et al. Associations between several sites of cancer and occupational exposure to benzene, toluene, xylene, and styrene: results of a case–control study in Montreal. Am J Ind Med. 1998;34(2):144–156. 9651624 10.1002/(sici)1097-0274(199808)34:2<144::aid-ajim7>3.0.co;2-x

[29] Warden H, Richardson H, Richardson L, et al. Associations between occupational exposure to benzene, toluene and xylene and risk of lung cancer in Montréal. Occup Environ Med. 2018;75(10):696–702. 29764994 10.1136/oemed-2017-104987

[30] Gashimova E, Temerdashev A, Porkhanov V, et al. Investigation of different approaches for exhaled breath and tumor tissue analyses to identify lung cancer biomarkers. Heliyon. 2020;6(6):e04224. 32577579 10.1016/j.heliyon.2020.e04224 PMC7305397

[31] O'Neill HJ, Gordon SM, O'Neill MH, et al. A computerized classification technique for screening for the presence of breath biomarkers in lung cancer. Clin Chem. 1988;34(8):1613–1618. 3042190

[32] Poli D, Carbognani P, Corradi M, et al. Exhaled volatile organic compounds in patients with non-small cell lung cancer: cross sectional and nested short-term follow-up study. Respir Res. 2005;6(1):71–10. 16018807 10.1186/1465-9921-6-71 PMC1185565

[33] Song G, Qin T, Liu H, et al. Quantitative breath analysis of volatile organic compounds of lung cancer patients. Lung Cancer. 2010;67(2):227–231. 19409642 10.1016/j.lungcan.2009.03.029

[34] Corradi M, Poli D, Banda I, et al. Exhaled breath analysis in suspected cases of non-small-cell lung cancer: a cross-sectional study. J Breath Res. 2015;9(2):027101. 25634546 10.1088/1752-7155/9/2/027101

[35] Kamissoko A, Carré V, Schramm S, et al. Study of the mainstream cigarette smoke aerosols by Fourier transform ion cyclotron resonance mass spectrometry coupled to laser/desorption and electrospray ionization – additional insights on the heteroaromatic components. Rapid Commun Mass Spectrom. 2019;33(S1):95–108. 30440095 10.1002/rcm.8353

[36] Wu F, Yin Z, Yang L, et al. Smoking induced extracellular vesicles release and their distinct properties in non-small cell lung cancer. J Cancer. 2019;10(15):3435–3443. 31293647 10.7150/jca.30425 PMC6603414

[37] Bao L, Zhang Y, Wang J, et al. Variations of chromosome 2 gene expressions among patients with lung cancer or non-cancer. Cell Biol Toxicol. 2016;32(5):419–435. 27301951 10.1007/s10565-016-9343-z

[38] Chen C, Fu X, Zhang D, et al. Varied pathways of stage IA lung adenocarcinomas discovered by integrated gene expression analysis. Int J Biol Sci. 2011;7(5):551–566. 21552421 10.7150/ijbs.7.551 PMC3088877

[39] Asgari Y, Khosravi P, Zabihinpour Z, et al. Exploring candidate biomarkers for lung and prostate cancers using gene expression and flux variability analysis. Integr Biol (Camb). 2018;10(2):113–120. 29349465 10.1039/c7ib00135e

[40] Lim YW, Evangelista JS, Schmieder R, et al. Clinical insights from metagenomic analysis of sputum samples from patients with cystic fibrosis. J Clin Microbiol. 2014;52(2):425–437. 24478471 10.1128/JCM.02204-13 PMC3911355

[41] Koukourakis MI, Giatromanolaki A, Sivridis E, Tumor and Angiogenesis Research Group, et al. Pyruvate dehydrogenase and pyruvate dehydrogenase kinase expression in non small cell lung cancer and tumor-associated stroma. Neoplasia. 2005;7(1):1–6. 15736311 10.1593/neo.04373 PMC1490315

[42] Sellers K, Fox MP, Bousamra M, et al. Pyruvate carboxylase is critical for non-small-cell lung cancer proliferation . J Clin Invest. 2015;125(2):687–698. 25607840 10.1172/JCI72873 PMC4319441

[43] Feng H-M, Zhao Y, Zhang J-P, et al. Expression and potential mechanism of metabolism-related genes and CRLS1 in non-small cell lung cancer. Oncol Lett. 2018;15(2):2661–2668. 29434989 10.3892/ol.2017.7591 PMC5777290

[44] Kumar N, Shahjaman M, Mollah MNH, Bioinformatics Lab, Department of Statistics, Rajshahi University, Rajshahi, Bangladesh, et al. Serum and plasma metabolomic biomarkers for lung cancer. Bioinformation. 2017;13(06):202–208. 28729763 10.6026/97320630013202 PMC5512859

[45] Campanella A, De Summa S, Tommasi S. Exhaled breath condensate biomarkers for lung cancer. J Breath Res. 2019;13(4):044002. 31282387 10.1088/1752-7163/ab2f9f

[46] Zou Y, Wang L, Zhao C, et al. CEA, SCC and NSE levels in exhaled breath condensate—possible markers for early detection of lung cancer. J Breath Res. 2013;7(4):047101. 24185583 10.1088/1752-7155/7/4/047101

[47] Bikov A, Hernadi M, Zita Korosi B, et al. Expiratory flow rate, breath hold and anatomic dead space influence electronic nose ability to detect lung cancer. BMC Pulmonary Med. 2014;14(1):1–9. 10.1186/1471-2466-14-202 PMC4289562 25510554

