# Supplementary material for: Exhaled metabolic markers and relevant dysregulated pathways of lung cancer: a pilot study
Source: Ann Med. 2022 Mar 9;54(1):790–802. doi: 10.1080/07853890.2022.2048064 (PMC8920387; doi:10.1080/07853890.2022.2048064)
Supplement: Supplemental Material [file IANN_A_2048064_SM8363.zip › Supplemental files/revised supplementary materials.docx]

**S1 Inclusion criteria and exclusion criteria**

The inclusion criteria of LC cases were as follows: all cases 1) were confirmed with an incident of LC histologically or pathologically and 2) had a pulmonary function test with a FEV1/FVC score larger than 70 % (FEV1: forced expiratory volume in 1 s, FVC: forced vital capacity). The exclusion criteria were as follows: 1) patients have received treatment, such as chemotherapy and surgical operation, after the diagnosis of LC; 2) patients had a history of airway inflammatory or lung infection in the past 3 months; 3) patients had a previous cancer; 4) patients who did not understand or cooperate with any part of the gas collection process.

The healthy controls were matched to the LC patients on the basis of age, gender and smoking history. The inclusion criteria of controls were as follows: all controls 1) were confirmed without clinical symptoms of LC (hemoptysis, cough, dyspnea, weight loss and so on); 2) had negative results of LDCT scans and 3) had a pulmonary function test with a FEV1/ FVC score larger than 70 %. The exclusion criteria were as follows: 1) controls had a history of airway inflammatory and lung infection in the past 3 months; 2) controls had a previous cancer; 3) controls who did not understand or cooperate with any part of the gas collection process.

**S2 Details of data acquisition**

**Sample collection:** Clinical information about the subjects is concealed from the laboratory staff members who performed the breath collection and assay. Subjects were asked to have a 12-hour fasting and rinse out their mouth with distilled water before breath collection. During collection, subjects were asked to breathe tidally into the self-developed collection device. VOCs were captured and concentrated into a Tenax TA stainless steel tube (PerkinElmer, Waltham, MA, USA). Due to its low affinity for water, Tenax TA is especially useful for the purging and trapping of volatiles from high moisture content samples including the analysis of VOCs in breath. PerkinElmer claimed the sealed Tenax TA stainless steel tube can preserve the exhaled breath sample for a year. That is to say, the time of shipping among hospital and lab did not affect the results of analysis. Using the self-made collection device, we could collect alveolar breath and remove dead space breath with the help of a three-way valve and flow meter. The whole process took about 5 min and 1000 mL exhaled breath could been concentrated. Repeated tests on samples of air and human breath were performed to ensure the consistency of the analysis procedures.

Chemical analysis: The chemical analysis was performed on GC-MS (QP2010 Plus, Shimadzu, Tokyo, Japan) coupled with a thermal desorption (TD) instrument (TurboMatrix 300 TD, PerkinElmer, Waltham, MA, USA). TD was used to desorb the VOCs adsorbed in the Tenax TA stainless steel tube. Desorption was performed under a 10 mL/min helium flow at 250 ◦C for 10 min. The volatiles were then concentrated in a cold trap at − 30 ◦C and transferred directly onto the head of the capillary column by heating the cold trap to 250 ◦C (at a rate of 40 ◦C/s) and held for 3 min. The outlet split was set at a ratio of 1:5. Then VOCs were sent to GC-MS, and the temperature of the injector was set to 250 ℃. VOCs were separated on an Rtx-5 column (30.0 m Length * 0.25 mm ID * 0.25 μm Thickness, Restek). The column temperature program was initially 40℃ maintained for 1 min, then ramped at 5 ℃/min to 250℃ and held for 2 min. The MS was in scan mode in the 45–500 mass to charge (m/z) range. The scan time was 2.5–38 min. The temperatures of the electron impact ion source and interface were set at 200 ℃ and 250 ℃, respectively. The solvent cutoff time was 0.4 min.

**Calibration:** Prior to the use, all glassware were cleaned with methanol and dried in an oven at 60 ℃ overnight. Tedlar bags were filled with high purity nitrogen and heated to 60 ℃ in the oven. After held for 30 min, we pumped out those nitrogen and repeat these steps three times. A standard gas sample of 2,5-dimethylfuran which appeared in 80 % detected beath samples was used in calibration experiments. 2,5-Dimethylfuran (99 %, Aladdin, China) was dissolved in methanol (99.5 %, Sinopharm chemical Reagent Co., Ltd., Shanghai, China). Gaseous standards were prepared by injecting the mixed solution into the gas generator (MF-3B, China National Metrology Technology Development Co., Beijing, China). The concentrations of 2,5-dimethylfuran in the mixed gas were 0.1, 0.2, 0.5, 1.0, and 2.0 ppb, which covered the range in exhaled gas. Gaseous standards were kept in the prior cleaned Tedlar bag (SKC, USA). The process of analysis was as same as that for exhaled breath. Results indicated the detection system had a good linear response (R2 = 0.9984, p < 0.0001).

**GC-MS data pretreatment:** The spectrum analysis was performed using GC-MS Solution (Shimadzu, Tokyo, Japan). An initial list of peaks was generated using the semi-automated tool (GC-MS Postrum Analysis) in GC-MS Solution package. Peak identification requires a half-height peak width less than 2 s, a slope of 100/min, and a peak area larger than the limit of quantification (LOQ) which is defined as 10 times of standard deviation. Peaks were identified by spectral match according to the mass spectrometry library NIST 05 and NIST 05 s. Data extracted from each peak, including retention time (RT), CAS No., chemical identity, area under curve (AUC), and quality of fit, need to be confirmed by researchers. After that they were automatically downloaded into a text file. Peak AUC assigned the same RT was extracted to construct a two-dimension table using our self-developed computer program (VOC analysis V1.0, Registration No.: 2015SR129892). A single sample was used to generate the initial list of peaks, and additional peaks encountered were added to the list. Peaks having the same CAS No. and time difference of RTs less than 0.3 min were regarded as the same feature in machine learning model. Each sample was then vectorized by the AUCs of those extracted peaks which appear at least once in samples. Ambient air was collected and analyzed according to the procedure described above. Background components were removed by subtracting vector of ambient air from that of breath sample. Breath profiles based on those differential vectors, representing as rows, were merged together to form a two-dimensional dataset for following analysis.

**S3 Power analysis plot**


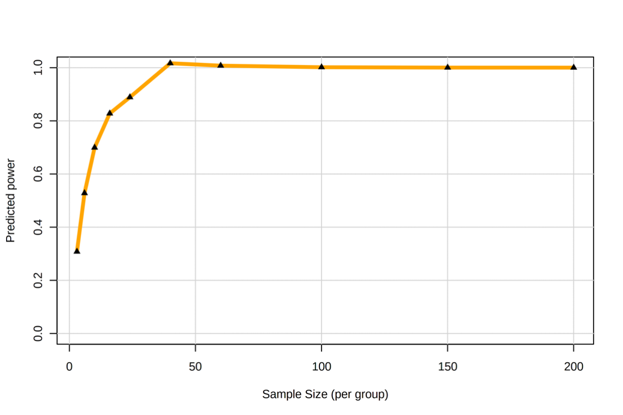


Results of power analysis, when FDR were set as 0.05. The predicted power increases fast when sample size smaller than 40, and it tends to stabilize after that. Therefore, the number of subjects in each group should be larger than 40.

**S4 Group-wise differential metabolites**

| **Subjects** | **Metabolites** | **HMDB ID** | **log_2_(FC)** | **p.ajusted** |
| --- | --- | --- | --- | --- |
| Males | 2,5-Dimethylfuran | HMDB0033182 | -4.7679 | 1.56E-05 |
|  | 3-(Methylthio)-1-propene | HMDB0031653 | -2.3099 | 7.85E-06 |
|  | 3-Ethyltoluene | HMDB0059848 | -2.1737 | 5.33E-08 |
|  | 3-Carene | HMDB0035619 | -2.1201 | 0.00069803 |
|  | 1-Methylnaphthalene | HMDB0032860 | -1.4436 | 0.055501 |
|  | Acetic acid | HMDB0000042 | -1.3575 | 0.004127 |
|  | 3,3-Dimethylhexane | HMDB0031418 | -1.3237 | 0.0031257 |
|  | Limonene | HMDB0032473 | -1.1053 | 0.0050572 |
|  | n-Octanal | HMDB0001140 | 1.0156 | 0.026689 |
|  | 3-Methylbutanol | HMDB0006007 | 1.2022 | 5.62E-06 |
|  | n-Nonane | HMDB0029595 | 1.3125 | 0.0031257 |
|  | 1-Octene | HMDB0032449 | 1.6521 | 0.011496 |
|  | n-Nonanal | HMDB0059835 | 1.7249 | 0.0012912 |
|  | n-Octanol | HMDB0001183 | 3.0479 | 0.001488 |
|  | Hexanal | HMDB0005994 | 3.1843 | 0.00089531 |
|  | 2-Butoxyethanol | HMDB0031327 | 3.5187 | 0.0012912 |
|  | Eucalyptol | HMDB0004472 | 5.3517 | 0.004127 |
| Females | 3-Methylheptane | HMDB0031583 | -4.9367 | 0.064486 |
|  | 3-(Methylthio)-1-propene | HMDB0031653 | -4.7648 | 0.036142 |
|  | o-Cymene | HMDB0037050 | -3.5339 | 0.068672 |
|  | 3-Ethyltoluene | HMDB0059848 | -2.1213 | 0.0016931 |
|  | n-Octanal | HMDB0001140 | 1.3583 | 0.064486 |
|  | o-Xylene | HMDB0059851 | 1.7734 | 0.096529 |
|  | Hexanal | HMDB0005994 | 2.4146 | 0.0082685 |
|  | Phytol | HMDB0002019 | 2.5024 | 0.05291 |
|  | n-Butylacetate | HMDB0031325 | 4.3466 | 0.030914 |
|  | Propyl acetate | HMDB0034237 | 4.4434 | 0.041594 |
| Smokers | Isopropylbenzene | HMDB0034029 | -6.136 | 0.06886 |
|  | 2,5-Dimethylfuran | HMDB0033182 | -4.8701 | 1.94E-06 |
|  | 3-(Methylthio)-1-propene | HMDB0031653 | -3.2045 | 5.45E-07 |
|  | 3-Ethyltoluene | HMDB0059848 | -2.2195 | 8.63E-08 |
|  | 3-Carene | HMDB0035619 | -2.1868 | 0.0016116 |
|  | 1-Methylnaphthalene | HMDB0032860 | -1.7239 | 0.060935 |
|  | Acetic acid | HMDB0000042 | -1.583 | 0.0073325 |
|  | 3,3-Dimethylhexane | HMDB0031418 | -1.5144 | 0.010716 |
|  | 3-Methylheptane | HMDB0031583 | -1.2583 | 0.021621 |
|  | o-Xylene | HMDB0059851 | -1.2218 | 3.82E-05 |
|  | 2-Ethylhexanol | HMDB0031231 | 1.0761 | 0.076729 |
|  | n-Octanal | HMDB0001140 | 1.0925 | 0.010716 |
|  | Propyl acetate | HMDB0034237 | 1.1112 | 0.06103 |
|  | n-Undecane | HMDB0031445 | 1.3066 | 0.073743 |
|  | 1-Octene | HMDB0032449 | 1.5712 | 0.0016116 |
|  | n-Nonane | HMDB0029595 | 1.7782 | 0.00012858 |
|  | n-Nonanal | HMDB0059835 | 1.9098 | 0.001402 |
|  | 3-Methylbutanol | HMDB0006007 | 2.5936 | 1.30E-05 |
|  | n-Octanol | HMDB0001183 | 3.1767 | 0.0016116 |
|  | Hexanal | HMDB0005994 | 3.5172 | 0.001739 |
|  | 2-Butoxyethanol | HMDB0031327 | 4.9236 | 0.0015984 |
|  | Eucalyptol | HMDB0004472 | 5.894 | 0.010716 |
| Non-smokers | l-Menthol | HMDB0003352 | -3.7234 | 0.069658 |
|  | 3-Carene | HMDB0035619 | -3.4067 | 0.069658 |
|  | 3-(Methylthio)-1-propene | HMDB0031653 | -2.8056 | 0.073311 |
|  | 3-Ethyltoluene | HMDB0059848 | -1.787 | 0.00050329 |
|  | Dimethyldisulfide | HMDB0005879 | -1.4166 | 0.081296 |
|  | o-Xylene | HMDB0059851 | 2.0048 | 0.081296 |
|  | Hexanal | HMDB0005994 | 2.1666 | 0.0026525 |
|  | n-Butylacetate | HMDB0031325 | 4.6661 | 0.063266 |
| All subjects | 2,5-Dimethylfuran | HMDB0033182 | -4.9137 | 1.63E-06 |
|  | Isopropylbenzene | HMDB0034029 | -4.2289 | 0.030046 |
|  | 3-(Methylthio)-1-propene | HMDB0031653 | -3.0083 | 1.88E-08 |
|  | 3-Carene | HMDB0035619 | -2.6349 | 2.00E-05 |
|  | o-Cymene | HMDB0037050 | -2.0044 | 6.35E-05 |
|  | 3-Ethyltoluene | HMDB0059848 | -1.9675 | 3.02E-12 |
|  | 3-Methylheptane | HMDB0031583 | -1.7361 | 0.001547 |
|  | l-Menthol | HMDB0003352 | -1.5572 | 0.011647 |
|  | 1-Methylnaphthalene | HMDB0032860 | -1.5146 | 0.046745 |
|  | Camphene | HMDB0059839 | -1.3967 | 0.004879 |
|  | 4-Isopropyltoluene | HMDB0005805 | -1.3903 | 0.035386 |
|  | Limonene | HMDB0032473 | -1.1501 | 0.000858 |
|  | Acetic acid | HMDB0000042 | -1.1047 | 0.00111 |
|  | Dimethyldisulfide | HMDB0005879 | -1.0665 | 0.000334 |
|  | n-Octanal | HMDB0001140 | 1.1063 | 0.001547 |
|  | n-Nonanal | HMDB0059835 | 1.256 | 0.00018 |
|  | 3-Methylbutanol | HMDB0006007 | 1.2972 | 3.90E-08 |
|  | 1-Octene | HMDB0032449 | 1.5238 | 0.004979 |
|  | Propyl acetate | HMDB0034237 | 2.066 | 0.004879 |
|  | 2-Butoxyethanol | HMDB0031327 | 2.312 | 0.000139 |
|  | n-Octanol | HMDB0001183 | 2.3218 | 0.017385 |
|  | n-Butylacetate | HMDB0031325 | 2.6097 | 0.011918 |
|  | n-Heptane | HMDB0031447 | 2.7725 | 0.010201 |
|  | Hexanal | HMDB0005994 | 2.9582 | 3.46E-06 |

**The two tables displaying correlation coefficients (S5) and p-values (S6) of correlation analysis were separately saved in two sheets of an independent xlsx file.**

**S7 Correlations of metabolites larger than 0.5**

| Metabolite A | | Metabolite B | | r | p-value |
| --- | --- | --- | --- | --- | --- |
| Name | HMDB ID | Name | HMDB ID |  |  |
| n-Butylacetate | HMDB0031325 | Propyl acetate | HMDB0034237 | 0.560 | <0.001 |
| 3-Methylbutanol | HMDB0006007 | 3-Ethyltoluene | HMDB0059848 | 0.519 | <0.001 |
| 2-Butoxyethanol | HMDB0031327 | 3-Ethyltoluene | HMDB0059848 | 0.566 | <0.001 |
| 2-Butoxyethanol | HMDB0031327 | 3-Methylbutanol | HMDB0006007 | 0.554 | <0.001 |
| o-Xylene | HMDB0059851 | n-Butylacetate | HMDB0031325 | 0.509 | <0.001 |
| o-Xylene | HMDB0059851 | 3-Ethyltoluene | HMDB0059848 | 0.535 | <0.001 |
| n-Nonanal | HMDB0059835 | n-Octanal | HMDB0001140 | 0.674 | <0.001 |

**S8 PLS-DA score plots**


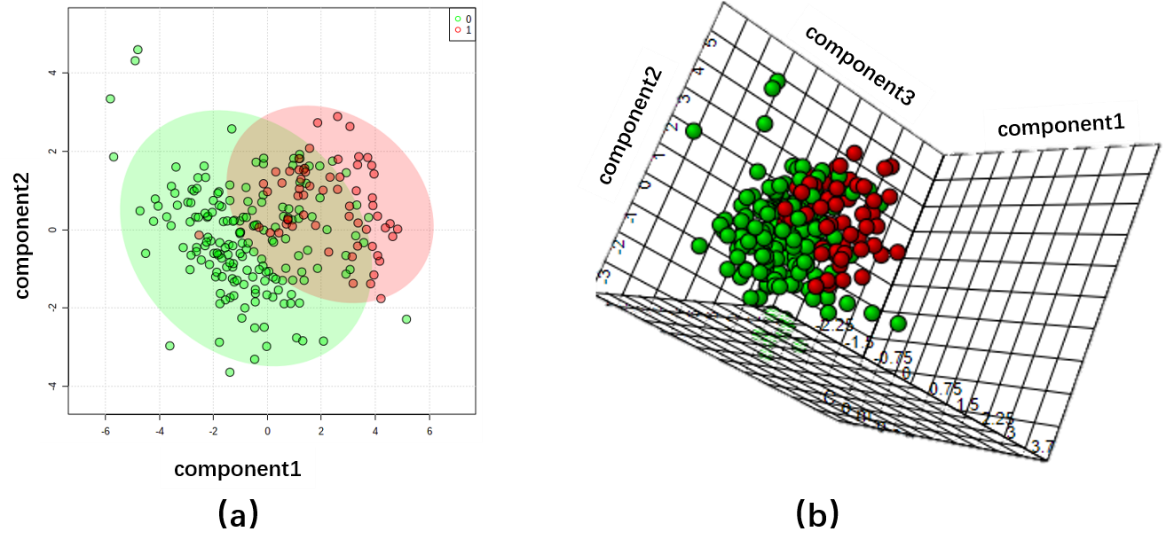


Results of PLS-DA based on 236*31 dataset. (a) 2D-Score plot, (b)3 D-Score plot. Green points representing controls while red points representing cases.
